# Molecular testing stratifies the risk of structural recurrence in high risk differentiated thyroid cancer: a retrospective cohort study

**DOI:** 10.3389/fendo.2025.1508404

**Published:** 2025-01-24

**Authors:** Jie Liu, Wensi Gao, Xiong Zheng, Shuping Wu, Yi Shi, Feng Wang, Yu Wu

**Affiliations:** ^1^ Department of Head and Neck Surgery, Clinical Oncology School of Fujian Medical University, Fujian Cancer Hospital, Fuzhou, China; ^2^ Department of Breast Surgery, Clinical Oncology School of Fujian Medical University, Fujian Cancer Hospital, Fuzhou, China; ^3^ Department of Molecular Pathology, Clinical Oncology School of Fujian Medical University, Fujian Cancer Hospital, Fuzhou, China

**Keywords:** thyroid cancer, risk stratification, molecular testing, recurrence, molecular profile

## Abstract

**Background:**

High-risk differentiated thyroid cancer in 2015 American Thyroid Association risk stratification system (ATA-RSS) exhibits a significantly increased probability of recurrence and poor outcomes. This study aimed to investigate the molecular profiles of high-risk differentiated thyroid cancer and to assess the role of molecular testing in enhancing prognostic risk stratification.

**Methods:**

In a single-center study conducted at Fujian Cancer Hospital, Fujian Province, China, a consecutive cohort of differentiated thyroid cancer patients identified as high-risk under 2015 ATA-RSS criteria were retrospectively assessed, spanning from November 1, 2019, to March 31, 2022. Molecular characterize groups were conducted using an 18-gene next-generation sequencing assay. Patients harboring mutations in the *TERT* promoter, *TP53*, or *PIK3CA* genes were categorized as the high molecular risk group, while all others were assigned to the non-high molecular risk group.

**Results:**

Among the 108 cases, 32 (29.6%) fell into the high molecular risk group, characterized by a significantly older mean age (57.8 vs. 42.6 years, *p* < 0.001), larger tumor size (3.1 cm vs. 2.0 cm, *p* = 0.003), a higher incidence of aggressive pathological subtypes (43.8% vs. 7.9%, *p* < 0.001), and an increased occurrence of distant metastasis (34.4% vs. 7.9%, *p* = 0.001). Over a median follow-up period of 32.5 months, this high-risk group demonstrated an elevated risk of local recurrence (32.1% vs. 9.5%, HR: 3.18, 95% CI: 1.15-8.78) and metachronous distant metastasis (38.1% vs. 2.9%, HR: 12.54, 95% CI: 2.60-60.41). Multivariate COX regression analysis confirmed that molecular characterize groups (HR: 5.77, 95% CI: 2.18-15.23, *p* < 0.001) and tumor size (HR: 1.32, 95% CI: 1.00-1.74, *p* = 0.047) independently predicted recurrence-free survival.

**Conclusion:**

ATA-RSS high-risk differentiated thyroid cancer often presents with late-hit genetic alterations, which are strongly associated with increased likelihood of structural recurrence. Molecular testing offers a precise approach to recurrence risk stratification in high-risk cases, enabling personalized follow-up and treatment strategies tailored to the specific prognostic profile.

## 1 Introduction

Thyroid cancer has demonstrated the most rapid rise in incidence among endocrine malignancies globally in recent decades. By 2020, the global incidence surpassed 500,000 new cases annually, with over 200,000 in China alone (1, 2). Despite differentiated thyroid cancer (DTC) comprising the majority of cases, often associated with favorable outcomes and low mortality, a subset of patients will experience relapse or metastasis, developing resistance to radioactive iodine (RAI), thus creating a substantial therapeutic obstacle (3). Postoperative management of DTC now primarily relies on risk-based assessments, prioritizing individualized approaches and ongoing adjustments. Prognostic evaluation is currently guided by the 8th edition of the American Joint Committee on Cancer Tumor-Node-Metastasis (AJCC TNM) staging system and the 2015 American Thyroid Association risk stratification system (ATA RSS), both of which are widely adopted methodologies (4). The 8th edition of the AJCC TNM staging system provides a more precise reflection of mortality risk. However, for evaluating postoperative recurrence risk in DTC patients with favorable prognoses, greater reliance on supplementary risk assessment tools is warranted (5). The 2015 ATA RSS serves as a key prognostic framework following DTC surgery. In a study with a median follow-up of 4 years, 31% of patients in the ATA high-risk group achieved no evidence of disease (NED), compared to 52% in the intermediate-risk group and 78% in the low-risk group (6). A separate short-term study reported recurrence rates of 22.8%, 6.7%, and 2.9% for the high-, intermediate-, and low-risk groups, respectively (7). These findings indicate that the ATA RSS stratifies recurrence risk with greater precision. In various regions, including China, the ATA RSS remains a foundational tool for postoperative risk assessment. However, the ATA RSS framework omits several critical individual variables, such as pathological subtypes, multifocality, extranodal invasion, and molecular profiles, resulting in substantial prognostic variability even among cases classified within the same risk group (4). Continued research and accumulating evidence are required to refine and enhance the accuracy of this stratification system. In the three risk classes, persistent and recurrent lesions frequently occur in high-risk DTC cases, posing significant obstacles to effective treatment (8, 9). Further investigation into prognostic factors for patients within this risk category remains essential.

Research into the molecular mechanisms of thyroid cancer has evolved over several decades, with early studies identifying key mutations in *BRAF* and *RAS*, along with fusion events in the *RET* and *NTRK* genes (10, 11). These genetic alterations critically affect the clinical and pathological characteristics of thyroid cancer by activating the mitogen activated protein kinase (MAPK) and/or PI3K/AKT/mTOR signaling pathways. Such oncogene mutations are present throughout all stages of thyroid cancer and are now recognized as early drivers of tumorigenesis (12). Molecular diagnostic tools that detect common early driver genes, such as *BRAF* V600E, *RAS*, *RET*, and *PAX8*/*PPARG*, when combined with fine needle aspiration biopsy (FNAB), have been shown to substantially enhance diagnostic accuracy, a finding supported by numerous studies and widely accepted in the field (13–16). Some of these driver alterations, such as *BRAF* V600E, *RET*, *NTRK* fusion, and *ALK* fusion, also serve as therapeutic targets for selective kinase inhibitors. Compared to multi-targeted kinase inhibitors, these agents are often preferred due to their superior efficacy and safety profiles, underscoring the heightened clinical value of molecular testing in managing aggressive and RAI-refractory thyroid cancers (17). Mutations in the PI3K/AKT/mTOR pathway, including *PIK3CA*, *AKT1* and *PTEN* are infrequent in thyroid cancer, though they can act as early driver mutations in certain RAS-like subtypes. More commonly, however, they co-occur with other driver mutations in advanced, poorly differentiated cases (12, 18). Additionally, mutations in the *TERT* promoter (*TERT*p) and tumor suppressor genes like *TP53* and *CNKN2A* frequently co-mutate with driver genes. These mutations are closely linked to adverse prognostic indicators, such as recurrence, metastasis, and iodine resistance. Importantly, they are regarded as late-stage mutations that drive thyroid cancer dedifferentiation and progression to high-grade or undifferentiated forms (12, 18). These previous findings have enabled prognostic stratification of certain thyroid cancers through the integration of specific genetic markers in molecular testing. In recent years, several studies have sought to elucidate the association between the molecular profiles of thyroid cancer and clinical risk and prognosis. Yip et al. conducted a case-control study analyzing the genetic variation in DTC patients with distant metastases, proposing molecular risk stratification based on these variations, with distinct groups showing differing predicted rates of distant metastasis (19). Liu et al. and Schumm et al. further validated the relationship between this molecular risk stratification and tumor recurrence through retrospective cohort studies (7, 20). Collectively, these studies have highlighted the feasibility of molecular testing in enhancing current risk assessment models and introduced novel perspectives for its application in thyroid cancer management.

The molecular profiles of ATA high-risk DTC patients and its association with prognosis remain unexplored. This retrospective cohort study analyzed the clinical characteristics and follow-up data of ATA high-risk individuals who underwent 18-gene next-generation sequencing (NGS) test, aiming to evaluate the potential of incorporating molecular profiles to refine postoperative recurrence risk stratification in this high-risk population.

## 2 Materials and methods

### 2.1 Patients and clinicopathologic data

**Figure 1:**
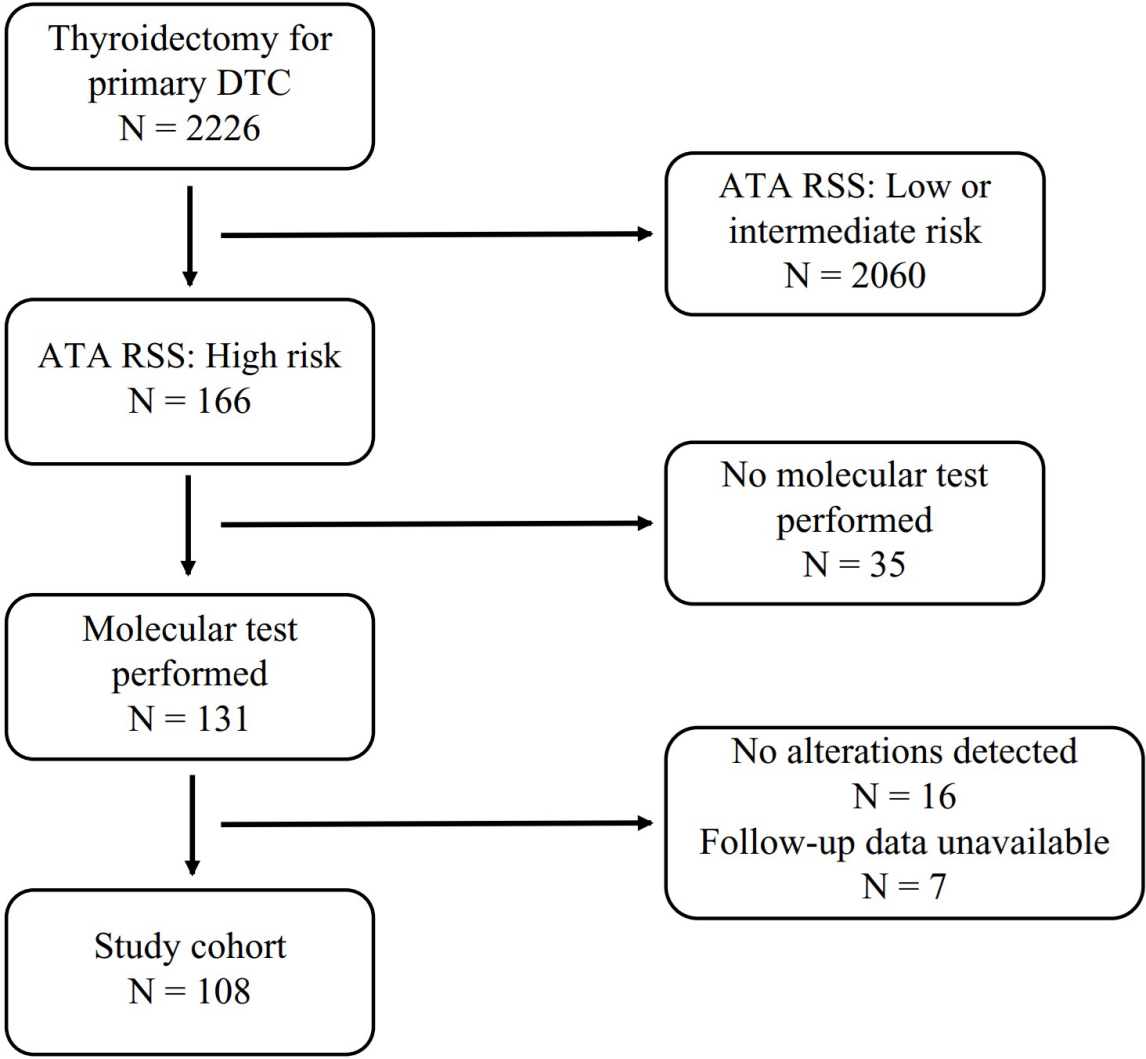
Flowchart of patient inclusion and exclusion. DTC, Differentiated Thyoid Cancer; ATA RSS, 2015 ATA Risk Stratification System.

This retrospective cohort study analyzed medical records from consecutive patients who underwent primary thyroidectomy with a pathological diagnosis of DTC at Fujian Cancer Hospital, Fujian Province, China, between November 1, 2019, and March 31, 2022, utilizing the hospital’s electronic medical record system. This study was approved by the Ethics Committee of Fujian Cancer Hospital, Fuzhou, China (Ethics Number: K2024-400-01). Preoperative assessment, surgical extent, and postoperative management adhered to the 2015 ATA guidelines and the Chinese National Health Commission’s thyroid cancer diagnosis and treatment protocols. The ATA high-risk category was assigned based on the criteria outlined in the 2015 ATA guidelines (4). This retrospective cohort study analyzed medical records from consecutive patients who underwent primary thyroidectomy with a pathological diagnosis of DTC at Fujian Cancer Hospital, Fujian Province, China, between November 1, 2019, and March 31, 2022, utilizing the hospital’s electronic medical record system. Preoperative assessment, surgical extent, and postoperative management adhered to the 2015 ATA guidelines and the Chinese National Health Commission’s thyroid cancer diagnosis and treatment protocols. The ATA high-risk category was assigned based on the criteria outlined in the 2015 ATA guidelines (**Figure 1**). Preoperative variables included patient age, sex, and tumor size. Imaging results from Doppler ultrasound, computed tomography (CT), and magnetic resonance imaging were assessed to verify the characteristics of the primary tumor, lymph node involvement, and distant metastasis. Surgical records were examined to determine macroscopic extrathyroidal extension (ETE) and extranodal extension (ENE) based on descriptions of primary tumors and regional lymph nodes. Postoperative pathology reports, reviewed by experienced pathologists, provided confirmation of pathological subtypes, lymph node metastasis, ETE, ENE, multifocality, and other relevant data. Notably, since molecular testing was conducted postoperatively, pathologists were unaware of these molecular findings at the time of initial pathological diagnosis of surgical specimens.

### 2.2 Molecular testing and molecular characterize groups

Molecular testing for thyroid tumors has not been routinely integrated into the diagnostic and therapeutic protocols in China. At our institution, such testing was recommended post-surgery for cases identified as high risk according to ATA RSS, where its potential clinical benefit was considered substantial. Patients were fully briefed on the associated costs and clinical implications, followed by informed consent if they opted for the testing. Sequencing libraries were constructed using the thyroid cancer 18-genes panel (RigenBio) in adherence to the manufacturer’s protocol. This panel, a multiplex PCR-based NGS assay, was designed to detect point mutations, insertions/deletions, and gene fusions across 18 genes implicated in thyroid cancer, including *AKT1*, *BRAF*, *CTNNB1*, *EZH1*, *GNAS*, *HRAS*, *KRAS*, *NRAS*, *NTRK*, *PAX8*/*PPARG*, *RET*, *PIK3CA*, *PTEN*, *SPOP*, *TERT*p, *TP53*, *TSHR*, and *ZNF148*. Genomic DNA and total RNA were extracted from formalin-fixed, paraffin-embedded (FFPE) thyroid nodule specimens utilizing the FFPE DNA/RNA Extraction Kit (RigenBio). Total RNA was reverse-transcribed into cDNA, and both DNA and cDNA were amplified via multiplex PCR targeting specific genomic regions. Each amplified library underwent indexing and adapter ligation through an additional round of PCR. Purified indexed libraries were quantified using a Qubit fluorometer (Thermo Fisher), and sequencing was performed on the NovaSeq 6000 platform (Illumina) with 150 bp paired-end reads. Adapter sequences at the 3’ and 5’ ends of the reads were trimmed using Trimmomatic (v0.38) prior to alignment. SNV and InDel calling was performed using VarScan (v2.3.9), and variants were annotated with VEP. Gene fusions were identified by analyzing fusion transcript sequences with a customized script. Following previously reported molecular risk stratification methods (7, 20–22) and integrating the molecular variation data from this study, molecular characterize groups (MCGs) were categorized based on missense mutations, insertions/deletions, and gene fusions identified in molecular testing. Cases with “negative” molecular testing results were excluded from the study cohort. Patients harboring any of the four gene mutations—*TERT*p, *TP53*, *PIK3CA*, and *AKT1*—were classified into the high molecular risk (HMR) group, while those without these mutations were assigned to the non-HMR group.

### 2.3 Postoperative management and follow-up

Postoperative assessments were conducted to classify pTNM stage followed the AJCC 8th edition. During follow-up, abnormal neck findings on ultrasound or CT were further evaluated using ultrasound-guided FNAB to confirm structural recurrence (SR). Local recurrence encompassed lymph node metastasis in the neck and upper mediastinum, as well as other thyroid-originating lesions in these regions. Metachronous distant metastasis was primarily identified through biochemical markers, including serum thyroglobulin and thyroglobulin antibodies, alongside imaging modalities such as CT and RAI whole-body scans. Final diagnoses were made by a multidisciplinary team consisting of specialists in thyroid surgery, endocrinology, radiotherapy, radiology, and pathology. The diagnosis of RAI-refractory disease adhered to the criteria specified in the 2015 ATA guidelines (4). The study assessed all-cause mortality using data from medical records and telephone follow-ups, with the endpoint defined as the date of the patient’s last follow-up or death. The follow-up records were concluded as of March 31, 2024.

### 2.4 Statistical analysis

Normally distributed continuous variables were presented as mean ± standard deviation, and inter-group differences were assessed via Student’s t-test. For non-normally distributed variables, data were expressed as median ± interquartile range (IQR), with the Mann-Whitney U test used for between-group comparisons. Categorical variables were evaluated using the χ² test, with Fisher’s exact test applied when necessary. Univariate logistic regression was employed to assess the association between MCGs and both local recurrence and metachronous distant metastasis. The Kaplan-Meier method was applied to assess the relationship between MCGs and recurrence-free survival (RFS). For the COX regression model, univariate regression analysis initially identified potential variables associated with RFS, focusing on established prognostic factors such as age (<55 or ≥55 years), gender, tumor size (cm), pathological subtype, multifocality, ETE, ENE, N stage, and RAI ablation (23, 24). To avoid omitting potential prognostic variables that could be correlated with the outcome in the multivariable analysis, we have relaxed the significance threshold for this step to p < 0.10, variables with *p* ≥ 0.10 were excluded. Multivariate analysis then incorporated the remaining variables from the univariate screening alongside MCGs. A proportional hazard regression model was constructed using the likelihood ratio test (Forward: LR) with maximum partial likelihood estimation to identify independent factors related to RFS. Statistical analysis and visualization were performed using IBM SPSS Statistics version 22.0 (IBM Corp., NY, USA). All statistical tests were two-sided, with *p* < 0.05 deemed statistically significant.

## 3 Results

### 3.1 Clinical features

In a cohort of 108 ATA high-risk DTC patients, 59 (54.6%) were female, with a mean age of 47.1 years (SD: 14.6) and a median tumor size of 2.1 cm (IQR: 1.3–4.0). The majority of patients (97.2%) underwent total thyroidectomy with concurrent cervical lymph node dissection, while 3 patients received lobectomy with central lymph node dissection. Postoperative pathology confirmed papillary thyroid carcinoma (PTC) in 105 cases (97.2%), of which 17 (15.7%) exhibited invasive features, including 16 tall cell and 1 columnar cell variant. The remaining 3 cases were identified as widely invasive follicular thyroid carcinoma (FTC). In this cohort, high-risk ATA criteria encompassed macroscopic ETE (93.5%), distant metastasis (14.8%), metastatic lymph nodes with a maximum diameter ≥3 cm (13.9%), incomplete tumor resection (5.6%), FTC exhibiting more than 4 vascular invasions (2.8%), and postoperative serum thyroglobulin (Tg) levels suggestive of distant metastasis (1.9%). Most cases presented with central (89.8%) or lateral neck (54.6%) lymph node metastasis. Among the 17 cases with initial distant metastasis, lung involvement predominated (82.4%), followed by bone metastasis (23.5%). Other metastatic sites included the abdomen and brain, with one case each (5.9%). Among the clinical characteristics, 48 cases (44.4%) presented with ENE, and 70 cases (64.8%) exhibited multifocal lesions. Three patients who initially underwent thyroid lobectomy did not proceed with the recommended total thyroidectomy. Postoperative assessment, which included imaging and RAI whole-body scans for cases with elevated Tg levels, identified 90 patients (83.3%) as NED. Of these, 71.1% underwent RAI ablation or adjuvant therapy as prescribed, while the remainder received only thyroid-stimulating hormone suppression therapy and routine follow-up. The 18 patients with unresectable locally persistent disease or distant metastasis were treated with therapeutic RAI.

### 3.2 Molecular profiles and molecular characterize groups

**Figure 2:**
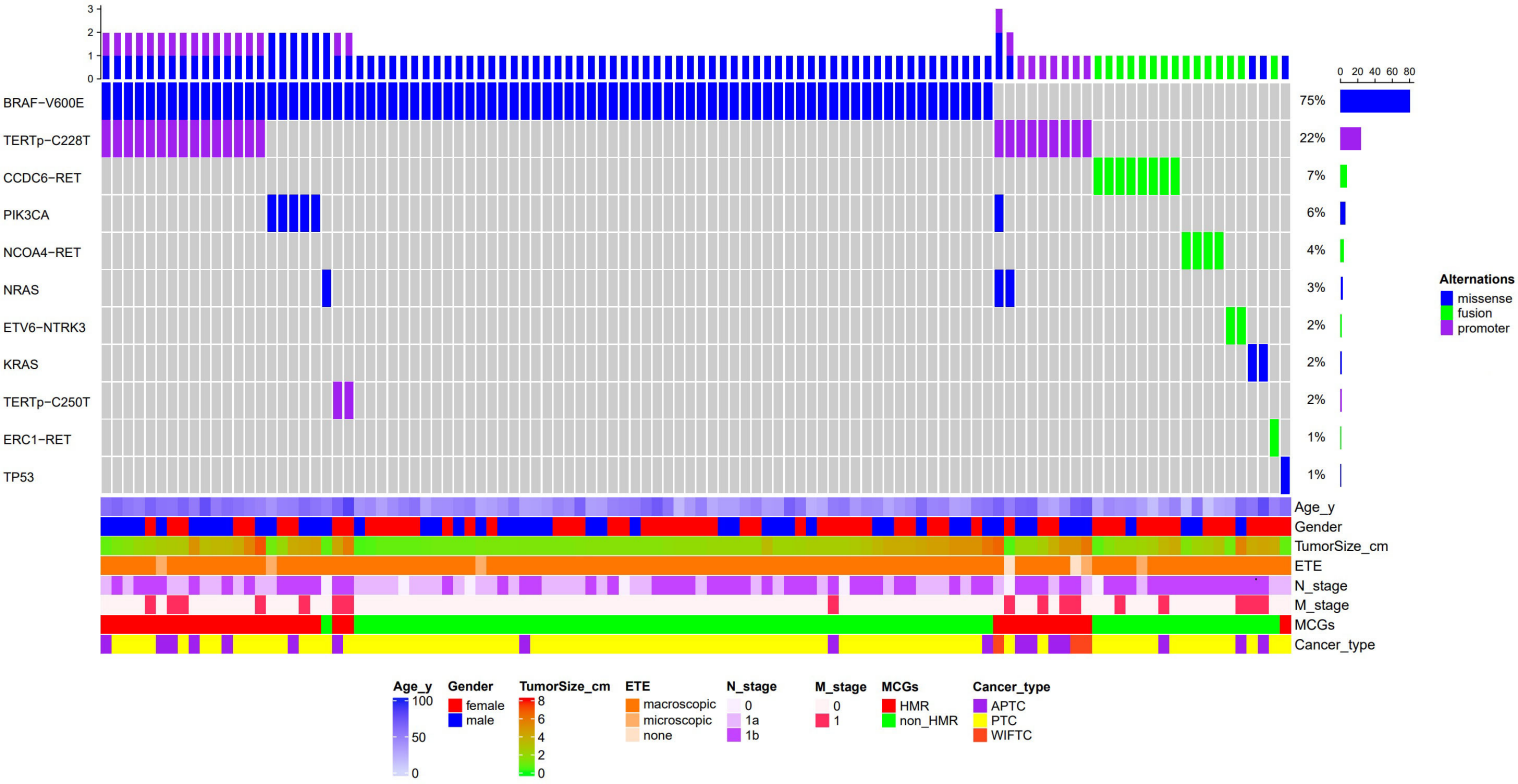
Waterfall plot of molecular profiles and clinical features of 108 patients with ATA high risk differentiated thyroid cancer. ETE, Extrathyroidal extension; MCGs, Molecular characterize groups; HMR, High Molecular Risk; APTC, aggressive papillary thyroid cancer; PTC, papillary thyroid cancer; WIFTC, widely invasive follicular thyroid cancer.

A total of 134 genetic variants across eight genes were identified in the study cohort, including 93 missense mutations, 26 upstream promoter mutations, and 15 fusion mutations, with the *BRAF* V600E mutation being the most prevalent (75%), followed by *TERT*p mutations (24.1%), *RET* fusions (12.0%), and *PIK3CA* mutations (5.6%) (**Figure 2**). Early driver mutations, such as *BRAF* V600E, *RET* fusion, *NTRK* fusion, and *KRAS*/*NRAS* mutations, were present in 92.6% of cases. Of the 8 cases with unidentified driver genes, 7 exhibited the single *TERT*p C228T mutation, while 1 presented with the single *TP53* E271K mutation. Based on the MCGs outlined earlier, 32 cases (29.6%) were assigned to the HMR group, and the remaining 76 cases were categorized as non-HMR. The HMR group demonstrated an older mean age (57.8 vs. 42.6, *p* < 0.001), more aggressive pathological subtypes (43.8% vs. 7.9%, *p* < 0.001), larger primary tumors (3.1 cm vs. 2.0 cm, *p* = 0.003), and a greater incidence of distant metastasis (34.4% vs. 7.9%, *p* = 0.001) (**Table 1**). Postoperatively, fewer patients in the HMR group achieved NED status (65.6% vs. 90.8%, *p =* 0.001). In cases achieving NED after initial treatment, 81.0% of the HMR group and 68.1% of the non-HMR group completed RAI ablation or adjuvant therapy, with no statistically significant difference observed between the groups (*p* = 0.256). A significant difference was found in ETE (*p =* 0.025), where 97.4% of the non-HMR group exhibited macroscopic ETE, compared to 84.4% in the HMR group. The female proportion was 40.6% in the HMR group and 60.5% in the non-HMR group, but this difference lacked statistical significance (*p* = 0.058). Regarding initial surgery, incomplete local tumor resection occurred in 12.5% of the HMR group and 2.6% of the non-HMR group, though this difference was also not statistically significant (*p* = 0.062).

**Table 1:** Demographic characteristics and clinical features of patients with ATA high risk differentiated thyroid cancer at initial surgery by Molecular Characterize Groups.

Variable		Molecular characterize groups, No. (%)		*p*
		Non-HMR	HMR	
No. of patients		76(70.4)	32(29.6)	
Age(Mean ± SD, years)		42.6 ± 13.4	57.8 ± 11.6	<0.001
SEX	Female	46(60.5)	13(40.6)	0.058
Cancer type	Non-Aggressive PTC,	70(92.1)	18(56.3)	<0.001
	Aggressive PTC	6(7.9)	11(34.4)	
	Widely invasive FTC	0	3(9.4)	
Initial surgery	Lobectomy and CND	2(2.6)	1(3.1)	0.959
	Total thyroidectomy and CND	33(43.4)	13(40.6)	
	Total thyroidectomy and LND	41(53.9)	18(56.3)	
	R2 resection	2(2.6)	4(12.5)	0.062
T stage	1	1(1.3)	2(6.3)	0.452
	2	1(1.3)	1(3.1)	
	3	30(39.5)	13(40.6)	
	4	44(57.9)	16(50.0)	
N stage	0	6(7.9)	1(3.1)	0.655
	1a	29(38.2)	13(40.6)	
	1b	41(53.9)	18(56.3)	
M stage	1	6(7.9)	11(34.4)	0.001
Extrathyroidal extension	Macroscopic	74(97.4)	27(84.4)	0.025
	Microscopic	2(2.6)	3(9.4)	
Extra-nodal invasion		31(40.8)	18(56.3)	0.204
Tumor size, median(IQR)		2.0(1.3-2.9)	3.1(2.0-4.5)	0.003
Multifocal		49(64.5)	21(65.6)	0.637
NED after initial surgery		69(90.8)	21(65.6)	0.001

ATA, American Thyroid Association; HMR, High Molecular Risk; PTC, papillary thyroid cancer; FTC, follicular thyroid cancer; CND, central neck dissection; LND, lateral neck dissection; NED, no evidence of disease.

### 3.3 Outcomes

**Figure 3:**
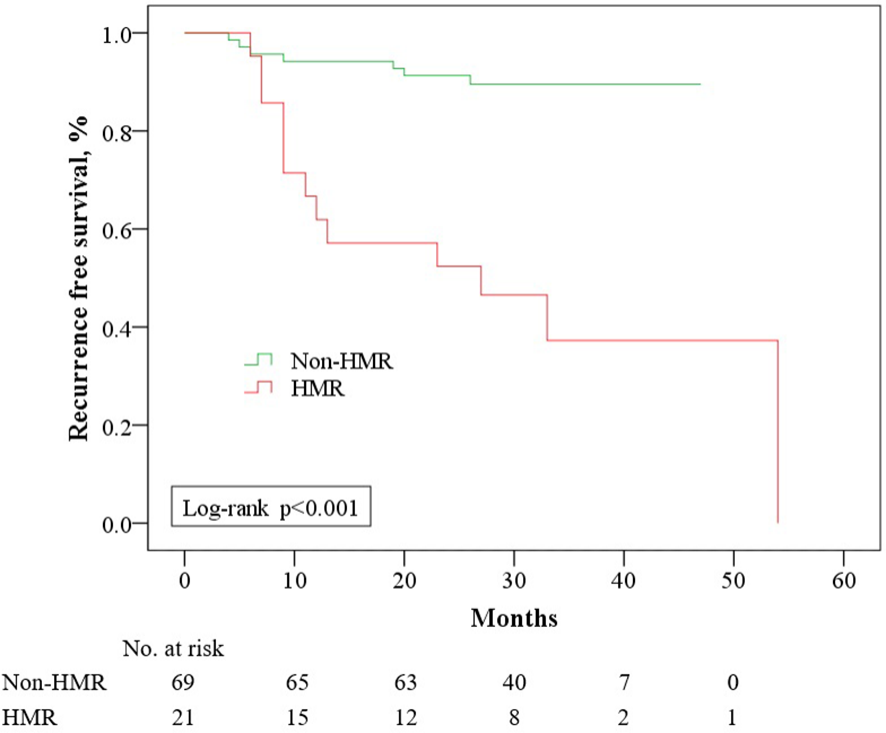
Recurrence free survival among 90 patients with ATA high risk differentiated thyroid cancer who had no evidence of disease after initial treatment, stratified by Molecular Characterize Groups. HMR, High Molecular Risk.

As of March 31, 2024, the median follow-up for the 108 cases was 32.5 months (IQR: 28–38), with no statistically significant difference in follow-up duration across groups (*p =* 0.159) (**Table 2**). In the HMR group, 17 cases (53.1%) and in the non-HMR group, 8 cases (10.5%) underwent therapeutic RAI for distant metastases or inoperable local lesions. Among these, 13 cases (76.5%) in the HMR group and 3 cases (37.5%) in the non-HMR group met RAI refractory criteria, although this difference did not reach statistical significance (*p =* 0.075). The HMR group exhibiting an elevated risk of local recurrence (HR: 3.18, 95% CI: 1.15-8.78) and metachronous distant metastasis (HR: 12.54, 95% CI: 2.60-60.41). Survival analysis indicated a strong association between MCGs and RFS (*p* < 0.001) (**Figure 3**). As described above, we first performed univariate analysis to preliminarily screen the initially identified variables, the results suggested that age, tumor size, N stage, and invasive pathological subtype may influence RFS (*p* < 0.10). Upon constructing the multivariate regression model, only MCGs (HR: 5.77, 95% CI: 2.18-15.23) and tumor size (HR: 1.32, 95% CI: 1.00-1.74) emerged as independent factors associated with RFS. During the follow-up period, 3 patients (9.4%) in the HMR group died, with survival times of 7, 15, and 22 months, respectively, all deaths attributable to the tumor. No deaths occurred in the non-HMR group, and the difference in mortality between the groups was statistically significant (*p =* 0.039).

**Table 2:** Outcome of patients with ATA high risk differentiated thyroid cancer by Molecular Characterize Groups.

Outcome		Molecular characterize groups, No. (%)		*p*
		Non-HMR	HMR	
Duration of follow-up, month, median(IQR)		32.5(27.0-36.0)	32.5(30.3-44.0)	0.159
Structural recurrence	Locally recurrence^1^	7(9.5)	9(32.1)	0.012
	Metachronous distant metastasis^2^	2(2.9)	8(38.1)	<0.001
Death		0	3(9.4)	0.039
RAI	Ablation or adjuvant therapy	47(68.1)	17(81.0)	0.256
	Therapeutic RAI	8(10.5)	17(53.1)	<0.001
	RAI-refractory disease	3(37.5)	13(76.5)	0.075

^1^2 patients in Non-HMR group and 4 patients in HMR group who had locally persistent disease were not included.

^2^6 patients in Non-HMR group and 11 patients in HMR group who had distant metastasis at presentation were not included.

ATA, American Thyroid Association; HMR, High Molecular Risk; RAI, radioactive iodine.

## 4 Discussion

ATA high-risk cases in DTC pose significant treatment challenges and are associated with poorer outcomes. Research on these high-risk cases remains limited, with considerable variation in reported characteristics across studies. A Dutch single-center study by Van Velsen et al., involving 236 ATA high-risk DTC patients, identified FTC in 32% of cases, ETE in 32%, cervical lymph node dissection in 45%, and distant metastasis in 33% (19). In contrast, a retrospective single-center study by Shah et al. in the United States, analyzing 320 ATA high-risk DTC cases, reported FTC in 4.1%, ETE in 94%, cervical lymph node metastasis (N1a+N1b) in 62.5%, and distant metastasis in 13.4%. The cohort characteristics observed in this study align with those reported by Shah et al., with a minimal incidence of FTC, most complicated by ETE, and 14.8% presenting with distant metastasis. These variations likely stem from differences in the pathological composition. In contrast, the higher proportion of FTC in van Velsen et al.’s study may explain the lower rates of ETE and lymph node metastasis, alongside a higher incidence of distant metastasis. Notably, the rate of cervical lymph node metastasis in this study exceeded that of the two aforementioned studies. Potential explanations include: (1) China’s diagnostic and treatment protocols still recommend routine prophylactic central cervical lymph node dissection, leading to the detection of subclinical metastases in cases, including those initially staged as cN0; (2) this study employed a more positive pre-treatment evaluation strategy for lateral cervical lymph nodes. For suspicious lesions detected on ultrasound or CT, ultrasound-guided FNAB combined with Tg measurement in puncture eluent enhanced the detection rate of lateral cervical lymph node metastasis (25). Additionally, 64.8% of the cases in this study involved multifocal lesions, with more than half (54.6%) exhibiting lateral cervical lymph node metastasis, and 44.4% showing ENE. These features align with previous reports on the characteristics of advanced DTC (3).

The NGS molecular assay used in this study incorporated an 18-gene panel, shown in prior research to improve diagnostic precision for FNAB (15). In contrast to widely adopted large NGS panel like ThyroSeq V3 and Afirma GSC, this assay targets the 18 most prevalent genes in Chinese thyroid cancer cases, optimizing cost-effectiveness. Previous studies employing large NGS panel reported variant detection rates ranging from 86.5% to 95.2% (20, 26, 27). In this study, clinically significant variants were identified in 87.8% of ATA high-risk DTC cases, with driver variants detected in 92.6%, reflecting comparable detection efficiency. Additionally, this molecular test covers several key prognosis-related genes, including *TERT*p, *TP53*, *PIK3CA*, *AKT1*, and *PTEN*, enhancing the ability to identify high-risk molecular profiles. Nevertheless, given the limited scope of molecular testing in this study, we believe that the molecular backgrounds of the “no alteration detected” cases remains unknown and may vary on an individual basis, which is why they were excluded from the study. Among the ATA high-risk cohort, the most prevalent driver variants were *BRAF* V600E, *NRAS*, *KRAS* mutations, and *RET* and *NTRK* fusions, aligning with findings from a recent Chinese study focused primarily on DTC (26). This distribution is also consistent with another report from China involving PTC cases with high recurrence risk (28). In this cohort, *TERT*p and *PIK3CA* mutations were identified in 24.1% and 5.6% of cases, respectively, notably exceeding the frequencies reported in other cohorts with varying recurrence risks. For instance, Du et al. reported *TERT*p and *PIK3CA* mutations in 6.3% and 1.5% of cases, respectively (26). Similarly, in a meta-analysis focusing solely on PTC, the average incidence of *TERT*p mutations was 10.1% (21). The elevated frequency of high-risk molecular alterations in the ATA high-risk DTC cohort suggests that prognostic gene detection in this group may hold enhanced clinical relevance. This study found that high-risk molecular alterations were consistently associated with *BRAF* V600E or *NRAS* mutations, while no such mutations were detected in the 15 cases exhibiting *RET* or *NTRK* fusions, aligning with findings from other large-scale studies. For instance, TCGA reports indicate that *TERT*p mutations are linked to driver mutations or arm-level somatic copy number alterations, rather than *BRAF* mutations or gene fusions (29). In the study by Du et al., only one case of *RET* fusion and one of *NTRK1* fusion were accompanied by *TERT*p mutations (26). However, other studies have noted the co-existence of *RET* fusions and *TERT*p mutations, which may impact prognosis and the response to *RET* and *MEK* targeted therapies (30). Further investigation into the association between gene fusion variants and late-stage alterations, such as *TERT*p mutations, is warranted as additional data becomes available.

A 2021 study by Yip et al. first proposed a novel molecular risk classification divided into three categories based on ThyroSeq, version 3 molecular testing and ThyroSeq Cancer Risk Classifier (19). This classification has since been adopted in several subsequent studies, with its clinical relevance consistently validated (7, 20, 22). The present study adapted this classification, modifying the risk grouping to align with the specific molecular tests used and the unique characteristics of the study cohort. Due to the limitations of molecular testing in this study in detecting certain early driver genes (e.g., *ALK*, *DICER1*, *EIF1AX*, etc.) and specific variant forms (e.g., gene expression alterations, copy number alterations, etc.), “concurrent early variants” were not considered a requirement for inclusion in the HMR group. Additionally, possibly influenced by the cohort characteristics, only two cases exhibited “RAS-like” molecular profiles, which were defined as “low molecular risk”. Consequently, the sample size for analyzing “low-risk” cases was insufficient, the non-HMR cases were not further stratified into “low-risk” or “intermediate-risk”. In the study by Liu et al., increasing molecular risk was associated with higher mean patient age, a lower proportion of female patients, larger median tumor size, and more frequent occurrences of aggressive PTC subtypes, ETE, cervical lymph node metastasis, and synchronous distant metastasis (22). Schumm et al. also identified a correlation between higher molecular risk groups and more aggressive clinical features, including larger primary tumors, ETE, and positive surgical margins (20). Similarly, in the HMR group of this study, clinical parameters such as age, tumor size, aggressive pathological subtypes, and distant metastasis demonstrated comparable patterns. Although *TERT*p mutations are well-documented as being significantly associated with RAI resistance (21, 31), the relationship between molecular risk stratification and RAI refractory status has not been explored. In this cohort, 25 patients underwent therapeutic RAI for unresectable lesions. While the HMR group exhibited a higher prevalence of RAIR cases, the difference was not statistically significant, likely due to the limited sample size. Further investigation is required to determine whether MCGs can reliably predict RAI treatment efficacy, with larger sample sizes necessary.

Previous studies have demonstrated a significant correlation between high-risk molecular variants and both RFS and SR following DTC surgery, with a strong link to distant metastasis. For example, Liu et al. observed a substantially lower 36-month RFS in the high molecular risk group compared to the intermediate and low-risk groups, with a recurrence risk exceeding threefold that of the latter two groups (22). Similarly, Schumm et al. found that patients in the high molecular risk group exhibited an elevated risk of SR (HR: 9.31) and distant metastasis (HR: 42.7) compared to those in the intermediate-risk group (20). In our study, a median follow-up of 32.5 months revealed significant differences in key prognostic indicators, including local recurrence, metachronous distant metastasis, and RFS across various MCGs following initial surgery. Notably, 80% of metachronous distant metastasis cases occurred in the HMR group. In contrast, the non-HMR group exhibited markedly lower rates of SR (10.1%) and metachronous distant metastasis (2.9%) compared to the overall study cohort, and significantly below historical data for ATA high-risk DTC (8, 9). All three recorded deaths occurred within the HMR group. Although between-group differences in mortality were significant, the limited follow-up duration and small number of death cases precluded survival analysis. These results indicate that for ATA high-risk DTC, the presence of high-risk variants serves as a strong predictor of adverse prognostic events during the initial treatment phase.

Some prognostic factors, such as age, tumor size, and pathological subtype, exhibit variability across different MCGs as previously noted, are currently employed to evaluate prognostic risk during the initial treatment phase of DTC (23). We hypothesized that high-risk genetic alterations contribute to the correlation between risk-related clinical features and poor prognosis, a relationship supported by emerging evidence. For instance, age is widely recognized as a key prognostic factor in DTC, as demonstrated in numerous studies (5, 32), and is a critical factor in the 8th AJCC TNM staging system. Nevertheless, research by Heo et al. found that *TERT*p mutations mediated the effect of age at diagnosis on the mortality rate by 36% in DTC (33). In this study, while age, tumor size, pathological subtype, and N stage appeared to be associated with RFS during initial univariate analysis, only MCGs (HR: 5.77) and tumor size (HR: 1.32) remained independently linked to RFS after incorporating the MCGs variable into a multivariable model. This finding aligns with reports by Liu et al. and Schumm et al., whose multivariate models similarly indicated that recurrence risk was solely related to tumor size and molecular risk (7, 20). Despite differences in cohort composition, the consistent results across these three DTC studies suggest that molecular profiles may be independently and more directly predictive of prognosis.

Several studies on “molecular risk stratification” have highlighted the importance of integrating routine molecular testing via preoperative FNAB, as it offers valuable insights into the molecular risk stratification prior to surgery, thereby shaping the initial treatment approach (7, 20, 22). However, despite its efficacy in informing clinical decisions, the broad adoption of NGS with large panels is hindered by substantial costs and limited access, restricting its routine application in many regions for thyroid cancer management. This study indicated that when NGS testing was employed for prognostic risk stratification in thyroid cancer, the use of small panels targeting key prognostic loci (such as *TERT*p, *PIK3CA*, *TP53*, *AKT1*, etc.) offered comparable efficiency in risk grouping. In regions where routine preoperative molecular testing is not feasible, a more targeted and cost-effective approach would be better to perform supplementary postoperative molecular testing specifically for ATA high-risk cases, allowing for accurate risk stratification. Given the elevated risk of recurrence and distant metastasis in patients with both high-risk ATA RSS and adverse molecular profiles, closer follow-up is warranted. Further research is needed to determine whether more positive RAI adjuvant therapies or alternative treatments may offer enhanced benefits for this subgroup.

Despite strengths, several limitations affect this study. First, as a retrospective cohort study, only cases with completed molecular testing were included, introducing potential selection bias due to the two-stage process of physician explanation and patient consent required for testing. Second, compliance issues led to incomplete RAI treatment in some cases, potentially skewing overall prognostic outcomes compared to patients who received the full standard treatment. Additionally, this non-compliance complicates a standardized analysis of postoperative serum Tg levels and biochemical incomplete responses within the study cohort. Given the lack of significant differences in case distribution across various MCG groups, this factor is unlikely to decisively affect the prognostic variation between the groups. Additionally, the small sample size limits the feasibility of performing propensity score matching based on different characteristics, which could have enhanced the precision of the statistical analysis. Furthermore, the short follow-up period constrains the study to short-term prognostic outcomes, and the long-term prognostic implications of different MCGs will require extended follow-up for more comprehensive assessment.

In conclusion, our study indicated a higher incidence of structural recurrence and poorer RFS in ATA RSS high risk DTC presented with high molecular risk. Molecular testing and MCGs offer significant potential for refining prognosis in high-risk DTC cases and merit broader application. The potential need for specialized postoperative management strategies for ATA high-risk cases with high molecular risk warrants further investigation.

## Data Availability

The datasets presented in this study can be found in online repositories. The names of the repository/repositories and accession number(s) can be found in the article/**Supplementary Material**.
